# Agreement between self-reported and general practitioner-reported chronic conditions among multimorbid patients in primary care - results of the MultiCare Cohort Study

**DOI:** 10.1186/1471-2296-15-39

**Published:** 2014-03-01

**Authors:** Heike Hansen, Ingmar Schäfer, Gerhard Schön, Steffi Riedel-Heller, Jochen Gensichen, Siegfried Weyerer, Juliana J Petersen, Hans-Helmut König, Horst Bickel, Angela Fuchs, Susanne Höfels, Birgitt Wiese, Karl Wegscheider, Hendrik van den Bussche, Martin Scherer

**Affiliations:** 1 Department of Primary Medical Care, Center of Psychosocial Medicine, University Medical Center Hamburg-Eppendorf, Martinistraße 52, 20246 Hamburg, Germany; 2 Department of Medical Biometry and Epidemiology, Center for Experimental Medicine, University Medical Center Hamburg-Eppendorf, Martinistraße 52, 20246 Hamburg, Germany; 3 Institute for Social Medicine, Occupational Health and Public Health, University of Leipzig, Semmelweisstraße 10, 04103 Leipzig, Germany; 4 Institute for General Practice, University of Jena, Bachstraße 18, 07743 Jena, Germany; 5 Central Institute of Mental Health, Medical Faculty Mannheim/Heidelberg University, Mannheim, Germany; 6 Institute for General Practice, University of Frankfurt am Main, Theodor-Stern-Kai 7, 60590 Frankfurt am Main, Germany; 7 Department of Health Economics and Health Services Research, University Medical Center Hamburg-Eppendorf, Martinistraße 52, 20246 Hamburg, Germany; 8 Institute of General Practice, Technical University of Munich, Ismaninger Straße 22, 81675 Munich, Germany; 9 Department of General Practice, University of Düsseldorf, Moorenstraße 5, 40225 Düsseldorf, Germany; 10 Department of Psychiatry and Psychotherapy, University of Bonn, Sigmund-Freud-Straße 25, 53105 Bonn, Germany; 11 Institute for Biometry, Hannover Medical School, 30623 Hannover, Germany

**Keywords:** Agreement, Self-report, Physician report, Chronic diseases, Primary care, Multimorbidity

## Abstract

**Background:**

Multimorbidity is a common phenomenon in primary care. Until now, no clinical guidelines for multimorbidity exist. For the development of these guidelines, it is necessary to know whether or not patients are aware of their diseases and to what extent they agree with their doctor. The objectives of this paper are to analyze the agreement of self-reported and general practitioner-reported chronic conditions among multimorbid patients in primary care, and to discover which patient characteristics are associated with positive agreement.

**Methods:**

The MultiCare Cohort Study is a multicenter, prospective, observational cohort study of 3,189 multimorbid patients, ages 65 to 85. Data was collected in personal interviews with patients and GPs. The prevalence proportions for 32 diagnosis groups, kappa coefficients and proportions of specific agreement were calculated in order to examine the agreement of patient self-reported and general practitioner-reported chronic conditions. Logistic regression models were calculated to analyze which patient characteristics can be associated with positive agreement.

**Results:**

We identified four chronic conditions with good agreement (e.g. diabetes mellitus κ = 0.80;PA = 0,87), seven with moderate agreement (e.g. cerebral ischemia/chronic stroke κ = 0.55;PA = 0.60), seventeen with fair agreement (e.g. cardiac insufficiency κ = 0.24;PA = 0.36) and four with poor agreement (e.g. gynecological problems κ = 0.05;PA = 0.10).

Factors associated with positive agreement concerning different chronic diseases were sex, age, education, income, disease count, depression, EQ VAS score and nursing care dependency. For example: Women had higher odds ratios for positive agreement with their GP regarding osteoporosis (OR = 7.16). The odds ratios for positive agreement increase with increasing multimorbidity in almost all of the observed chronic conditions (OR = 1.22-2.41).

**Conclusions:**

For multimorbidity research, the knowledge of diseases with high disagreement levels between the patients’ perceived illnesses and their physicians’ reports is important. The analysis shows that different patient characteristics have an impact on the agreement. Findings from this study should be included in the development of clinical guidelines for multimorbidity aiming to optimize health care. Further research is needed to identify more reasons for disagreement and their consequences in health care.

**Trial registration:**

ISRCTN89818205

## Background

In the future, more and more elderly people will require medical attention due to a chronic disease. Many older people even have multiple chronic conditions. This phenomenon is known as multimorbidity. Studies on multimorbidity showed repeatedly that there is no uniform definition of the term [1,2]. Some studies, for example, defined multimorbidity as the presence of two chronic diseases, while others required at least three chronic diseases. Various authors’ reviews showed a wide range in the prevalence of multimorbidity (3.5% to 98.5%) [1,3]. Not only the definition of multimorbidity, but also the data sources (patient reports, claims data or collected through physician or medical records) seem to have a decisive influence on the measured prevalence of multimorbidity [4-6]. Another problem concerning multimorbidity are the missing clinical guidelines for medical practice [7,8]. For the development of clinical guidelines, it is important to know if patients are aware of their diseases and how much they agree with their doctor regarding these illnesses.

Studies investigating the agreement of physician and patient information on the morbidity of patients showed an association of various patient characteristics with the concordance of morbidity data. For example, agreement decreased with old age, male gender, low education, comorbidity, depression, cognitive decline and hospitalization [9-14]. The physician-reported data was usually collected from the medical records. In previous studies, doctors were rarely asked directly about their patients and few studies included GPs for the collection of data on patient morbidity [15-17]. Often only diseases with high prevalence were compared. The present study examined the agreement between self-reported and general practitioner-reported chronic conditions among multimorbid patients in primary care. The associations of patient characteristics on the agreement values were also examined.

The following research questions were addressed:

1. To what extent is there agreement between self-reported and general practitioner-reported chronic conditions among multimorbid patients in primary care?

2. Which patient characteristics predict the agreement between self-reported and general practitioner-reported chronic conditions?

## Methods

### Study design

The following analyses are based on data from the MultiCare Cohort Study. This is a multicenter, prospective, observational cohort study with a total of 3,189 multimorbid patients, ages 65 to 85, from general practices in Germany. The study protocol has previously been published [18]. The study participants were recruited from 158 GP practices near eight study centers distributed across Germany (Bonn, Düsseldorf, Frankfurt/Main, Hamburg, Jena, Leipzig, Mannheim and Munich). In each practice we created a list of patients based on the GP’s electronic database. This list encompassed all patients who were born between 1.7.1923 and 30.6.1943 and consulted their GP at least once within the last completed quarter (i.e. 3 month period). From this list we randomly selected 50 patients with multimorbidity and contacted them for written, informed consent. Multimorbidity was defined as the coexistence of at least three chronic conditions from a list of 29 diseases published elsewhere [18]. Patients were excluded from the study if they were not regulars of the participating practices (i.e. in case of emergency consultation of the GPs), if they were unable to participate in the interviews (especially blindness and deafness), or if they were not able to speak and/or read German. Further exclusion criteria were: nursing home residency, severe illnesses (probably lethal within three months according to the GP), insufficient ability to consent (especially dementia) and participation in other studies at the present time [18]. The sampling and response rate have been published by Schäfer et al. [19]. In short, 24,862 patients were randomly selected from the study practices and checked for multimorbidity and exclusion criteria. 7,172 of these patients were eligible for study participation and contacted for informed consent to do so. Of all contacted patients, a total of 3,317 patients agreed to participate, which corresponds to a total response rate of 46.2%. Retrospectively we had to exclude 128 patients, because they passed away before the baseline interview or because we found out that the patients met exclusion criteria which their GP did not know of. Finally, 3,189 patients were included in the study. Recruitment and baseline data collection took place from July 2008 to October 2009.

### Data collection

A comprehensive description of the data sources, as well as the collected data, can be found in the study protocol [18]. Information regarding the patients’ diseases was collected in personal interviews with the patients and their GPs. The interviews were conducted by trained scientists and study nurses using a standardized list of defined diagnosis groups (based on ICD-10). The development of this list and the 46 diagnosis groups with corresponding ICD codes have been described elsewhere [20,21]. In short, the diagnosis groups are based on the most frequent conditions found in GP practices as mentioned: in a panel survey of the Central Research Institute of Statutory Ambulatory Health Care in Germany (“ADT-Panel”) [22], in the scientific experts’ report for the formation of a morbidity-orientated, risk adjustment scheme in the German Statutory Health Insurance [23], and in the data from the Gmünder ErsatzKasse (GEK) - a statutory health insurance company operating nationwide in Germany, insuring about 1.7 million people (2006) [21]. The ICD-10 system was used, because all issues managed by physicians within statutory ambulatory care have to be coded in ICD-10 and forwarded to the health insurance companies as regulated by German law in §295(1) SGB V and §44(3) of the Federal Collective Agreement within the statutory health insurance system in Germany [24].

The names of the diagnosis groups for the patient interviews were adapted to a more patient-friendly language. This translation was done by an expert team of general practitioners with practical experience from the Department of Primary Medical Care of the University Medical Center Hamburg-Eppendorf. When developing the diagnosis group names, terms were selected that are commonly used by the patients in their daily routines. Therefore, several different names and symptom descriptions were used.

The compilation of the diagnosis groups list was not finished by the beginning of the baseline interviews. This led to 7 of the 46 diagnosis groups not being part of the standardized GP questionnaire during the baseline interviews. This applied to chronic gastritis, insomnia, allergies, hypotension, sexual dysfunction and tobacco abuse. Dementia was not present in the baseline questionnaire, because it served as an exclusion criterion. In addition, some diagnosis groups were asked only in the GP interviews, as they were deemed to be too intimate for the patient interviews, or they were already being asked in other questionnaires e.g. depression (see below). This includes the diagnosis groups: obesity, liver disease, depression, severe hearing loss, somatoform disorders, urinary incontinence and anxiety. Therefore, this study compared the self-reported and the general practitioner-reported chronic conditions for a total of 32 diagnosis groups. The selection of the diagnosis groups is shown in Additional file 1.

### Data analysis

The prevalence proportions for the 32 diagnosis groups, the kappa coefficients (with a 95% CI) and the proportions of specific agreement were calculated in order to examine the agreement of patient-reported and general practitioner-reported diseases. The proportions of specific agreement are positive and negative agreement. Positive agreement (PA) is calculated with the following formula: PA = 2a/(2a + b + c) and negative agreement (NA) with the formula: NA = 2d/(2d + b + c) [25]. Assuming that the patients are not aware of their disease if they don’t give an answer, missing values in the patients’ self-reported list of diseases were converted to: “no disease reported”.

To analyze the associations of patient characteristics to the agreement of reported illnesses, we calculated logistic regression models in 26 diagnosis groups. Diagnosis groups with less than 50 cases per independent variable were excluded from the multivariate models. At this point we applied strict criteria because we wanted to ensure an adequate filling of cells. We excluded gynecological problems, urinary tract calculi, anemia, psoriasis, migraine/chronic headache and Parkinson’s disease because of their low prevalence (compare Additional file 1). The total positive agreement (i.e. both GP and patient say yes) was defined as the dependent variable of the logistic regression models. Independent variables were sex, age (in years), education (low vs. medium/high), income (in Euro), disease count (number of chronic conditions from the general practitioner interviews), depression (not depressed vs. depressed), health related quality of life (scaled from 0–100) and nursing care dependency (no nursing care dependency vs. nursing care dependency). The education levels were split into three groups (low, medium, high) according to the international CASMIN classification [26]. Patients’ income was reported as the net income per month after being adjusted to the patients’ household sizes [27]. We made a logarithmic transformation for the income variable because we assumed a non-linear association. We defined depression as a score higher than six on the geriatric depression scale (GDS) [28]. We assessed the health-related quality of life with the EQ visual analogue scale (EQ VAS) [29]. Nursing care dependency was considered present when a patient had a health-insurance company issued nursing dependency level.

A p-value of <0.05 was used as the criterion for statistical significance. Due to the exploratory approach of the study, the results should therefore be considered exploratory and the p-values should be interpreted cautiously.

The analyses were performed using IBM SPSS Statistics Version 19, Microsoft Office Excel 2003 and R (version 3.0.1) [30].

### Missing values

Missing values in the dataset arising from item non-response have been imputed in order to avoid any bias generated by listwise deletion subjects with missing values from the statistical analyses using the procedure hot deck imputation. This imputation procedure is described elsewhere in detail [19]. We imputed missing values in the following variables: income (12.4% missing values), self-rated health (0.3%), depression (0.4%) and nursing care dependency (0.7%). The variables age, gender, education and disease count did not contain any missing values. The imputation of missing values was performed with the R 2.13.0 package StatMatch.

### Ethical considerations

The study was approved by the Ethics Committee of the Medical Association of Hamburg (approval no. 2881). Participants signed a written, informed consent form to participate in the study.

## Results

### Characteristics of the study population: patients and GPs

The socio-demographic characteristics of the study’s participants (patients and GPs) are shown in Table 1. The mean age of the patients at the time of their baseline interviews was 74.4 years. 59.3% were female, 56.2% were married and 27.7% were widowed. The majority of participating study patients (62.3%) had a low level of education (CASMIN grade 1). Only 4.5% had a health-insurance company issued nursing dependency level. On average, the patients reported 7.6 chronic conditions, whereas the physicians diagnosed 6.3 chronic conditions. The mean age of the GPs at baseline interview was 50.2 years. 60.8% were male. The physicians had an average of 15 years of practice experience. Most of the participating GPs had comparably large practices considering 51.3% treated 1,000 or more patients in each quarter (three month period).

**Table 1:** Characteristics of the MultiCare Cohort Study sample at baseline

**Patients**		**n**
Age (at baseline interview): mean ± sd	74.4 ± 5.2 years	3,189
Gender:		3,189
- Male	40.7%	1,298
- Female	59.3%	1,891
Marital status:		3,188
- Never married	5.9%	188
- Married	56.2%	1,791
- Estranged (living in separate homes)	2.3%	72
- Divorced	8.0%	255
- Widowed	27.7%	882
Education (in CASMIN grade):		3,189
- Grade 1 (low)	62.3%	1,986
- Grade 2 (medium)	26.8%	856
- Grade 3 (high)	10.9%	347
Household-size adjusted net income per month: Mean ± sd	1,412.40 ± 704 €	2,793
Nursing dependency level:		3,168
- No nursing dependency	95.6%	3,027
- Dependency level 1	3.4%	107
- Dependency level 2	1.0%	31
- Dependency level 3	0.1%	3
Number of chronic conditions*: mean ± sd		3,189
- Based on the patient self-report	7.6 (3.2)	
- Based on the general practitioner report	6.3 (2.3)	
**GPs**		
Age (at baseline interview): mean ± sd	50.2 ± 7.7 years	158
Gender:		158
- Male	60.8%	96
- Female	39.2%	62
Years of practice: mean ± sd	15.0 ± 7.7 years	158
Number of patients treated in practice in each quarter:		158
- 1,000 and more patients	51.3%	81
- 750 thru 999 patients	24.7%	39
- 500 thru 749 patients	19.6%	31
- 499 thru less patients	4.4%	7
Number of physicians working in practice:		158
- 1	48.1%	76
- 2	32.9%	52
- 3	13.35	21
- 4	3.2%	5
- 5	1.9%	3
- 6	0.6%	1

n: number of observations, sd: standard deviation, *based on the list of 32 chronic conditions used for the comparison.

### Prevalence of diagnosis groups in patients’ self-reports and general practitioner reports

The prevalence proportions of the patients’ self-reported diagnoses and general practitioner-reported diagnoses are shown in Table 2. The biggest difference in the prevalence when comparing patient self-reported and physician reported diagnoses concerned dizziness (GPs: 7.7% vs. patients: 35.0%). Other major differences occurred in severe vision reduction (18.9% vs. 44.0%), joint arthrosis (43.3% vs. 66.5%) and neuropathies (14.7% vs. 35.6%) where the patients reported the diagnoses more frequently than their GPs. GPs often reported a higher prevalence of diseases that can be easily measured by laboratory values e.g. lipid metabolism disorders or diabetes mellitus.

**Table 2:** Prevalence and agreement of the 32 diagnosis groups: General practitioner reports vs. patient self-reports (n = 3,189)

		**Prevalence**		**Proportions of specific agreement**		**Kappa statistics**		
**No**	**Diagnosis group**	**General practitioner report% (n)**	**Patient self-report% (n)**	**Positive agreement (PA)**	**Negative agreement (NA)**	**Kappa**	**(95% CI)**	
1	Hypertension	77.9 (2,483)	72.3 (2,307)	0.89	0.67	0.56	0.53	0.60
2	Lipid metabolism disorders	58.5 (1,867)	45.8 (1,460)	0.69	0.66	0.36	0.33	0.39
3	Chronic low back pain	49.5 (1,577)	62.2 (1,984)	0.67	0.58	0.26	0.23	0.29
4	Joint arthrosis	43.3 (1,382)	66.5 (2,120)	0.66	0.59	0.29	0.26	0.32
5	Diabetes mellitus	37.6 (1,199)	31.1 (992)	0.87	0.93	0.80	0.78	0.82
6	Thyroid dysfunction	33.8 (1,077)	31.1 (992)	0.73	0.87	0.60	0.57	0.63
7	Chronic ischemic heart disease	31.4 (1,000)	30.3 (966)	0.68	0.86	0.54	0.51	0.57
8	Prostatic hyperplasia (n = 1,298)	27.9 (362)	39.4 (511)	0.50	0.75	0.26	0.21	0.31
9	Cardiac arrhythmias	26.9 (858)	33.0 (1,053)	0.64	0.85	0.49	0.45	0.52
10	Asthma/COPD	24.2 (771)	22.0 (700)	0.70	0.91	0.61	0.58	0.64
11	Lower limb varicosis	23.3 (742)	36.2 (1,155)	0.53	0.80	0.34	0.31	0.38
12	Osteoporosis	19.8 (632)	21.6 (690)	0.65	0.91	0.56	0.52	0.60
13	Severe vision reduction	18.9 (604)	44.0 (1,403)	0.47	0.76	0.28	0.25	0.31
14	Cancers	18.3 (584)	10.8 (343)	0.57	0.93	0.50	0.46	0.55
15	Hyperuricemia/Gout	17.3 (552)	16.8 (536)	0.44	0.89	0.33	0.29	0.37
16	Atherosclerosis/PAOD	16.7 (531)	11.1 (354)	0.42	0.91	0.34	0.29	0.38
17	Neuropathies	14.7 (469)	35.6 (1,136)	0.35	0.78	0.18	0.15	0.21
18	Intestinal diverticulosis	14.5 (462)	13.6 (435)	0.44	0.91	0.34	0.30	0.39
19	Cardiac insufficiency	13.1 (417)	17.2 (548)	0.36	0.89	0.24	0.20	0.29
20	Cerebral ischemia/Chronic stroke	11.8 (376)	13.9 (444)	0.60	0.94	0.55	0.50	0.59
21	Renal insufficiency	10.7 (340)	9.7 (308)	0.42	0.93	0.35	0.30	0.41
22	Cardiac valve disorders	9.4 (300)	9.9 (317)	0.41	0.94	0.35	0.30	0.40
23	Chronic cholecystitis/Gallstones	7.9 (251)	8.5 (272)	0.39	0.95	0.33	0.28	0.39
24	Dizziness	7.7 (246)	35.0 (1,115)	0.25	0.80	0.14	0.11	0.16
25	Hemorrhoids	7.5 (239)	22.8 (727)	0.24	0.87	0.15	0.11	0.18
26	Anemias	4.3 (136)	5.3 (170)	0.36	0.97	0.33	0.26	0.40
27	Rheumatoid arthritis/Chronic polyarthritis	4.2 (134)	12.9 (411)	0.32	0.94	0.27	0.22	0.32
28	Psoriasis	3.6 (116)	6.7 (213)	0.44	0.97	0.41	0.34	0.48
29	Migraine/chronic headache	3.5 (113)	6.1 (196)	0.34	0.97	0.31	0.24	0.38
30	Gynecological problems (n = 1,891)	3.4 (64)	13.1 (248)	0.10	0.92	0.05	0.01	0.10
31	Parkinson’s disease	1.9 (62)	2.1 (67)	0.73	0.99	0.72	0.64	0.81
32	Urinary tract calculi	1.8 (58)	3.9 (124)	0.27	0.98	0.26	0.17	0.34

*CI*, Confidence interval; *COPD*, Chronic obstructive pulmonary disease; *PAOD*, Peripheral arterial occlusive disease.

### Agreement between patient self-reported and general practitioner-reported diagnoses

The kappa statistics and the proportions of specific agreement are presented in Table 2.

The diagnosis groups diabetes mellitus, Parkinson’s disease, thyroid dysfunction and asthma/COPD had a good agreement according to the Altman classification of kappa coefficients (0.61-0.80) [31]. A moderate agreement was found in hypertension, osteoporosis, cerebral ischemia/chronic stroke, chronic ischemic heart disease, cancers, cardiac arrhythmia and psoriasis (0.41-0.60). The majority (17) of the diagnosis groups only had fair agreement (0.21-0.40). Neuropathies, hemorrhoids, dizziness and gynecological problems only showed a poor agreement (<0.20).

The positive agreement (PA) estimates the probability with which the GPs and the patients both report the chronic condition. The highest values were found for hypertension and diabetes mellitus with more than 0.80. Chronic cholecystitis/gallstones, anemia, cardiac insufficiency, neuropathies, migraine/chronic headache, rheumatoid arthritis/chronic polyarthritis, urinary tract calculi, dizziness and hemorrhoids had a lower probability for positive agreement (0.21-0.40). Gynecological problems showed a PA of only 0.1.

The negative agreement (NA) measures the probability with which both the GPs and the patients did not report the chronic condition. 25 chronic conditions show high values for negative agreement (>0.80) (compare Table 2). The lowest NA values were found for joint arthrosis (0.59) and chronic low back pain (0.58).

### Patient characteristics associated with the agreement of patient self-reported and general practitioner-reported diagnoses

26 logistic regression models, whose results are summarized in Table 3, show the association of certain patient characteristics with the positive agreement of the patient self-reported and general practitioner-reported diagnoses.

**Table 3:** Patient characteristics associated with positive agreement between patient self-reports and general practitioner reports

	**Hypertension**				**Lipid metabolism disorder**				**Chronic low back pain**			
	**OR**	**95%**	**CI**	**P-value**	**OR**	**95%**	**CI**	**P-value**	**OR**	**95%**	**CI**	**P-value**
**Sex (female)**	1.06	0.91	1.24	0.463	0.90	0.77	1.05	0.186	***1.97***	***1.68***	***2.32***	***0.000***
**Age (for 10 year intervals)**	***1.29***	***1.11***	***1.50***	***0.001***	***0.64***	***0.55***	***0.74***	***0.000***	***0.71***	***0.62***	***0.82***	***0.000***
**Education (medium/high)**	1.03	0.88	1.21	0.709	1.11	0.95	1.31	0.177	0.88	0.74	1.03	0.108
**Income (natural logarithm)**	0.91	0.76	1.09	0.312	1.13	0.95	1.35	0.171	1.04	0.87	1.25	0.664
**Disease count (for 3 disease intervals)**	**1.69**	**1.52**	**1.87**	**0.000**	***1.81***	***1.64***	***1.99***	***0.000***	***2.08***	***1.88***	***2.30***	***0.000***
**Depression (GDS >6)**	0.87	0.68	1.11	0.254	1.08	0.85	1.37	0.544	1.18	0.93	1.50	0.175
**EQ VAS (for 10 point intervals)**	0.97	0.93	1.02	0.226	1.02	0.98	1.07	0.281	***0.86***	***0.82***	***0.90***	***0.000***
**Nursing care dependency**	0.69	0.47	1.00	0.053	0.75	0.51	1.12	0.157	1.05	0.72	1.54	0.793
**n**	3189				3189				3189			
	**Severe vision reduction**				**Joint arthrosis**				**Diabetes mellitus**			
	**OR**	**95%**	**CI**	**P-value**	**OR**	**95%**	**CI**	**P-value**	**OR**	**95%**	**CI**	**P-value**
**Sex (female)**	1.12	0.91	1.37	0.302	***2.01***	***1.72***	***2.36***	***0.000***	***0.64***	***0.55***	***0.75***	***0.000***
**Age (for 10 year intervals)**	***1.42***	***1.17***	***1.72***	***0.000***	1.13	0.98	1.31	0.094	***0.72***	***0.62***	***0.84***	***0.000***
**Education (medium/high)**	***1.27***	***1.03***	***1.57***	***0.025***	0.99	0.84	1.16	0.876	0.86	0.73	1.01	0.070
**Income (natural logarithm)**	1.10	0.86	1.40	0.445	0.96	0.81	1.15	0.691	***0.83***	***0.69***	***0.99***	***0.043***
**Disease count (for 3 disease intervals)**	***1.68***	***1.50***	***1.89***	***0.000***	***1.69***	***1.54***	***1.87***	***0.000***	***1.48***	***1.35***	***1.63***	***0.000***
**Depression (GDS >6)**	1.12	0.83	1.53	0.456	0.81	0.63	1.02	0.078	1.06	0.83	1.36	0.643
**EQ VAS (for 10 point intervals)**	1.01	0.95	1.07	0.744	***0.91***	***0.87***	***0.95***	***0.000***	0.98	0.94	1.03	0.521
**Nursing care dependency**	0.79	0.48	1.30	0.353	0.88	0.61	1.28	0.501	1.29	0.89	1.88	0.180
**n**	3189				3189				3189			
	**Chronic ischemic heart disease**				**Thyroid dysfunction**				**Cardiac arrhythmias**			
	**OR**	**95%**	**CI**	**P-value**	**OR**	**95%**	**CI**	**P-value**	**OR**	**95%**	**CI**	**P-value**
**Sex (female)**	***0.29***	***0.24***	***0.34***	***0.000***	***4.51***	***3.67***	***5.54***	***0.000***	***0.65***	***0.54***	***0.79***	***0.000***
**Age (for 10 year intervals)**	1.05	0.88	1.25	0.608	***0.71***	***0.60***	***0.84***	***0.000***	***1.21***	***1.01***	***1.44***	***0.036***
**Education (medium/high)**	0.93	0.77	1.12	0.435	***1.28***	***1.07***	***1.53***	***0.007***	1.02	0.84	1.23	0.846
**Income (natural logarithm)**	0.86	0.69	1.06	0.161	1.15	0.94	1.41	0.186	1.06	0.85	1.31	0.611
**Disease count (for 3 disease intervals)**	***1.57***	***1.41***	***1.75***	***0.000***	***1.37***	***1.23***	***1.52***	***0.000***	***1.54***	***1.38***	***1.71***	***0.000***
**Depression (GDS >6)**	0.96	0.72	1.28	0.799	***0.65***	***0.48***	***0.86***	***0.003***	0.82	0.61	1.10	0.190
**EQ VAS (for 10 point intervals)**	***0.93***	***0.88***	***0.98***	***0.010***	1.01	0.96	1.07	0.640	0.95	0.90	1.00	0.071
**Nursing care dependency**	1.12	0.74	1.70	0.596	0.66	0.40	1.07	0.095	1.24	0.82	1.88	0.298
**n**	3189				3189				3189			
	**Hyperuricemia/Gout**				**Prostatic hyperplasia**				**Lower limb varicosis**			
	**OR**	**95%**	**CI**	**P-value**	**OR**	**95%**	**CI**	**P-value**	**OR**	**95%**	**CI**	**P-value**
**Sex (female)**	***0.50***	***0.38***	***0.66***	***0.000***					***2.61***	***2.08***	***3.27***	***0.000***
**Age (for 10 year intervals)**	0.93	0.71	1.21	0.571	***1.34***	***1.00***	***1.80***	***0.047***	1.17	0.97	1.42	0.103
**Education (medium/high)**	1.13	0.84	1.50	0.417	1.28	0.94	1.75	0.113	0.98	0.79	1.21	0.838
**Income (natural logarithm)**	1.03	0.74	1.42	0.866	1.00	0.70	1.42	0.986	1.01	0.80	1.28	0.940
**Disease count (for 3 disease intervals)**	***2.06***	***1.77***	***2.39***	***0.000***	***1.66***	***1.40***	***1.98***	***0.000***	***1.79***	***1.59***	***2.01***	***0.000***
**Depression (GDS >6)**	0.70	0.44	1.10	0.118	1.14	0.67	1.91	0.633	0.88	0.65	1.21	0.442
**EQ VAS (for 10 point intervals)**	0.93	0.86	1.00	0.066	1.06	0.97	1.16	0.172	1.03	0.97	1.09	0.317
**Nursing care dependency**	0.93	0.49	1.76	0.828	1.01	0.49	2.07	0.989	0.81	0.49	1.34	0.411
**Sex (female)**	3189				1298				3189			
	**Asthma/COPD**				**Atherosclerosis/PAOD**				**Osteoporosis**			
	**OR**	**95%**	**CI**	**P-value**	**OR**	**95%**	**CI**	**P-value**	**OR**	**95%**	**CI**	**P-value**
**Sex (female)**	0.92	0.76	1.12	0.410	***0.30***	***0.22***	***0.41***	***0.000***	***7.16***	***5.21***	***9.85***	***0.000***
**Age (for 10 year intervals)**	***0.72***	***0.59***	***0.87***	***0.001***	0.94	0.69	1.26	0.665	1.16	0.94	1.42	0.160
**Education (medium/high)**	1.03	0.84	1.26	0.780	***0.57***	***0.40***	***0.81***	***0.002***	***1.27***	***1.01***	***1.58***	***0.038***
**Income (natural logarithm)**	***0.67***	***0.54***	***0.84***	***0.001***	0.88	0.61	1.27	0.485	1.03	0.80	1.32	0.831
**Disease count (for 3 disease intervals)**	***1.30***	***1.16***	***1.45***	***0.000***	***1.61***	***1.36***	***1.90***	***0.000***	***1.22***	***1.07***	***1.38***	***0.003***
**Depression (GDS >6)**	1.07	0.80	1.43	0.649	***1.97***	***1.31***	***2.96***	***0.001***	0.83	0.60	1.14	0.252
**EQ VAS (for 10 point intervals)**	***0.87***	***0.82***	***0.92***	***0.000***	0.93	0.85	1.01	0.089	***0.90***	***0.84***	***0.95***	***0.001***
**Nursing care dependency**	0.65	0.40	1.07	0.091	1.06	0.57	1.98	0.855	1.56	0.99	2.46	0.056
**n**	3189				3189				3189			
	**Renal insufficiency**				**Cerebral ischemia/Chronic stroke**				**Cardiac insufficiency**			
	**OR**	**95%**	**CI**	**P-value**	**OR**	**95%**	**CI**	**P-value**	**OR**	**95%**	**CI**	**P-value**
**Sex (female)**	***0.35***	***0.24***	***0.51***	***0.000***	***0.51***	***0.39***	***0.67***	***0.000***	***0.69***	***0.49***	***0.97***	***0.032***
**Age (for 10 year intervals)**	***1.79***	***1.27***	***2.53***	***0.001***	1.17	0.90	1.52	0.234	***2.25***	***1.63***	***3.10***	***0.000***
**Education (medium/high)**	1.10	0.76	1.61	0.607	0.78	0.59	1.05	0.101	0.74	0.51	1.07	0.108
**Income (natural logarithm)**	1.00	0.65	1.55	0.995	***1.45***	***1.05***	***2.01***	***0.025***	0.70	0.48	1.04	0.080
**Disease count (for 3 disease intervals)**	***1.91***	***1.58***	***2.30***	***0.000***	***1.43***	***1.23***	***1.66***	***0.000***	***2.23***	***1.88***	***2.64***	***0.000***
**Depression (GDS >6)**	0.84	0.49	1.44	0.531	1.00	0.66	1.51	0.992	1.08	0.70	1.66	0.736
**EQ VAS (for 10 point intervals)**	***0.87***	***0.78***	***0.96***	***0.007***	1.00	0.93	1.09	0.945	***0.80***	***0.72***	***0.88***	***0.000***
**Nursing care dependency**	1.41	0.73	2.72	0.312	***5.23***	***3.40***	***8.06***	***0.000***	***1.75***	***1.02***	***3.00***	***0.043***
**Sex (female)**	3189				3189				3189			
	**Chronic cholecystitis/Gallstones**				**Hemorrhoids**				**Intestinal diverticulosis**			
	**OR**	**95%**	**CI**	**P-value**	**OR**	**95%**	**CI**	**P-value**	**OR**	**95%**	**CI**	**P-value**
**Sex (female)**	***1.86***	***1.18***	***2.93***	***0.007***	0.75	0.51	1.09	0.133	***2.07***	***1.49***	***2.88***	***0.000***
**Age (for 10 year intervals)**	***1.58***	***1.07***	***2.34***	***0.020***	0.69	0.47	1.01	0.058	***0.72***	***0.54***	***0.97***	***0.031***
**Education (medium/high)**	0.74	0.47	1.16	0.190	1.41	0.95	2.09	0.087	1.15	0.84	1.57	0.378
**Income (natural logarithm)**	0.76	0.47	1.22	0.255	0.83	0.54	1.28	0.402	1.34	0.94	1.92	0.107
**Disease count (for 3 disease intervals)**	***1.58***	***1.27***	***1.98***	***0.000***	***2.41***	***1.98***	***2.94***	***0.000***	***1.90***	***1.61***	***2.23***	***0.000***
**Depression (GDS >6)**	0.57	0.28	1.19	0.133	1.32	0.76	2.27	0.322	0.79	0.48	1.29	0.340
**EQ VAS (for 10 point intervals)**	1.04	0.92	1.18	0.487	1.07	0.96	1.20	0.216	0.99	0.91	1.09	0.900
**Nursing care dependency**	0.55	0.17	1.83	0.330	1.19	0.53	2.70	0.670	0.38	0.14	1.09	0.071
**n**	3189				3189				3189			
	**Rheumatoid arthritis/Chronic polyarthritis**				**Cardiac valve disorders**				**Neuropathies**			
	**OR**	**95%**	**CI**	**P-value**	**OR**	**95%**	**CI**	**P-value**	**OR**	**95%**	**CI**	**P-value**
**Sex (female)**	***2.81***	***1.65***	***4.79***	***0.000***	1.00	0.69	1.45	0.986	***0.71***	***0.55***	***0.92***	***0.009***
**Age (for 10 year intervals)**	0.77	0.50	1.17	0.219	1.22	0.87	1.73	0.252	***0.75***	***0.58***	***0.97***	***0.026***
**Education (medium/high)**	1.46	0.93	2.29	0.102	0.90	0.62	1.33	0.609	1.13	0.86	1.48	0.374
**Income (natural logarithm)**	1.29	0.77	2.15	0.338	1.05	0.68	1.61	0.839	1.00	0.74	1.35	0.994
**Disease count (for 3 disease intervals)**	1.02	0.78	1.34	0.870	***1.48***	***1.21***	***1.81***	***0.000***	***1.98***	***1.72***	***2.27***	***0.000***
**Depression (GDS >6)**	0.61	0.31	1.20	0.152	1.16	0.67	2.01	0.587	***1.45***	***1.02***	***2.07***	***0.036***
**EQ VAS (for 10 point intervals)**	***0.72***	***0.64***	***0.82***	***0.000***	1.03	0.93	1.15	0.583	***0.89***	***0.83***	***0.96***	***0.003***
**Nursing care dependency**	1.23	0.50	3.01	0.656	0.40	0.12	1.32	0.135	1.58	0.96	2.59	0.070
**n**	3189				3189				3189			
	**Dizziness**				**Cancers**							
	**OR**	**95%**	**CI**	**P-value**	**OR**	**95%**	**CI**	**P-value**				
**Sex (female)**	***1.45***	***1.02***	***2.05***	***0.037***	***0.66***	***0.51***	***0.86***	***0.002***				
**Age (for 10 year intervals)**	***1.38***	***1.01***	***1.88***	***0.040***	0.93	0.73	1.20	0.596				
**Education (medium/high)**	0.98	0.69	1.39	0.899	1.15	0.88	1.49	0.313				
**Income (natural logarithm)**	1.47	0.99	2.20	0.059	1.29	0.95	1.74	0.101				
**Disease count (for 3 disease intervals)**	***1.99***	***1.68***	***2.35***	***0.000***	1.02	0.87	1.19	0.849				
**Depression (GDS >6)**	***1.98***	***1.32***	***2.98***	***0.001***	0.85	0.56	1.30	0.457				
**EQ VAS (for 10 point intervals)**	***0.89***	***0.81***	***0.98***	***0.018***	0.97	0.90	1.05	0.483				
**Nursing care dependency**	0.64	0.31	1.32	0.224	***2.67***	***1.65***	***4.33***	***0.000***				
**n**	3189				3189							

*n*, Number of cases; *CI*, Confidence interval; *OR*, Odds ratio; *COPD*, Chronic obstructive pulmonary disease; *PAOD*, Peripheral arterial occlusive disease, statistically significant results (p < 0.05) in italic and bold letters.

Women had higher odds ratios for a positive agreement with their GP regarding the diagnosis groups: osteoporosis (OR = 7.16), thyroid dysfunction (OR = 4.51), rheumatoid arthritis/chronic polyarthritis (OR = 2.81), varicosis (OR = 2.61), intestinal diverticulosis (OR = 2.07), joint arthrosis (OR = 2.01), chronic low back pain (OR = 1.97), chronic cholecystitis/gallstones (OR = 1.86) and dizziness (OR = 1.45). The association with positive agreement was lower for chronic ischemic heart disease (OR = 0.29), atherosclerosis/PAOD (OR = 0.30), renal insufficiency (OR = 0.35), hyperuricemia/gout (OR = 0.50), cerebral ischemia/chronic stroke (OR = 0.51), diabetes mellitus (OR = 0.64), cardiac arrhythmias (OR = 0.65), cancers (OR = 0.66), cardiac insufficiency (OR = 0.69) and hemorrhoids (OR = 0.71).

Age was associated with positive agreement for cardiac insufficiency (OR = 2.25), renal insufficiency (OR = 1.79), chronic cholecystitis/gallstones (OR = 1.58), severe vision reduction (OR = 1.42), dizziness (OR = 1.38), prostatic hyperplasia (OR = 1.34), hypertension (OR = 1.29) and cardiac arrhythmias (OR = 1.21). The association with positive agreement was lower for lipid metabolism disorders (OR = 0.64), thyroid dysfunction (OR = 0.71), chronic low back pain (OR = 0.71), diabetes mellitus (OR = 0.72), intestinal diverticulosis (OR = 0.72), asthma/COPD (OR = 0.72) and neuropathies (OR = 0.75). The odds ratios for age were calculated using ten year intervals.

Patients with a medium or high education had higher odds ratios for positive agreement, compared to patients with low education, for the diagnosis groups thyroid dysfunction (OR = 1.28), severe vision reduction (OR = 1.27) and osteoporosis (OR = 1.27). This association was lower for atherosclerosis/PAOD (OR = 0.57).

The natural logarithm of household-size adjusted, net income showed an association with positive agreement for three chronic conditions: asthma/COPD (OR = 0.67), diabetes mellitus (OR = 0.83) and cerebral ischemia/chronic stroke (OR = 1.45). The odds ratios for income were calculated using one step on the logarithmic scale. One step, for example, including 400 € to 1,100 € net income per month, another step covering 3,000 € to 8,100 € per month.

The disease count showed higher odds ratios for positive agreement for 24 diagnosis groups. The odds ratios, calculated for a difference of three diseases, were between 1.22 and 2.41. The highest odds ratios were found for hemorrhoids (OR = 2.41), cardiac insufficiency (OR = 2.23), chronic low back pain (OR = 2.08) and hyperuricemia/gout (OR = 2.06).

Patients, with a potential depression according to the GDS, had lower association of positive agreement for thyroid dysfunction (OR = 0.65). The odds ratios were higher for dizziness (OR = 1.98), atherosclerosis/PAOD (OR = 1.97) and neuropathies (OR = 1.45).

The effect of the EQ visual analogue scale was calculated in 10 point intervals on the scale of 0–100. We found a lower association with positive agreement for chronic rheumatoid arthritis/chronic polyarthritis (OR = 0.72), cardiac insufficiency (OR = 0.80), chronic low back pain (OR = 0.86), asthma/COPD (OR = 0.87), renal insufficiency (OR = 0.87), neuropathies (OR = 0.89), dizziness (OR = 0.89), osteoporosis (OR = 0.90), joint arthrosis (OR = 0.91) and chronic ischemic heart disease (OR = 0.93).

Patients with a nursing care dependency had higher odds ratios for positive agreement regarding cerebral ischemia/chronic stroke (OR = 5.23), cancers (OR = 2.67) and cardiac insufficiency (OR = 1.75).

## Discussion

### Agreement between patient self-reports and general practitioner reports

The results of our analysis indicate that the agreement of self-reported and general practitioner-reported chronic conditions in a multimorbid elderly population is dependent on the type of disease and varies by the patients’ characteristics.

Very good agreement was found for diabetes mellitus. Many other studies also showed very good or good agreement for this illness [10,11,13,14,16,17,32-38]. Generally, a very good agreement between patients and their GPs for diabetes mellitus is not surprising. The treatment of diabetes mellitus requires a high level of patient participation: regular blood glucose monitoring, dietary changes, etc. In addition, many patients in Germany with diabetes mellitus are enrolled in a disease management program (DMP) that requires regular appointments and prescribed examinations, so that both physicians and patients are often confronted with the disease. The same could apply to the diagnosis group asthma/COPD, for which there is also a DMP in Germany. The agreement on asthma/COPD was good in our cohort. Other studies also found good and moderate agreement for asthma [14,33,35]. However, Merkin et al. found only poor agreement for COPD, in a study with end-stage renal patients [34].

Moderate agreement, based on kappa statistics and high PA, was found for hypertension. Many other studies confirm this result [11,13,16,35,37-40]. In many cases, medication is needed, as well as regular blood pressure monitoring. High prevalence in the elderly population, drug treatments and regular follow-up appointments may have a positive effect on the agreement of GP and patient. However, studies which include younger people indicate poor to fair kappa coefficients for hypertension [33,34].

We found moderate agreement for the diagnosis group cerebral ischemia/chronic stroke. Other studies reported higher values [9,11,13,32,34], while Muggah et al. reported merely a fair agreement for stroke in their study on the comparison of health-administrative data and patient self-report which included patients ages 20 to over 75 [38]. The differences, in comparison to the majority of the other studies, could be explained by a specific cohort or lower number of cases. For instance, the study participants of Bush et al. were also participants of a screening program for elderly patients [32]. Due to the screening program participants may have greater attention for their illness and for the accurate reporting of their diseases in the study. In the MultiCare Cohort Study only 12.5% of all patients with cerebral ischemia/chronic stroke had a nursing care dependency, therefore, the lesser disease severity could explain the poorer concordance. In addition, ischemic stroke often results in dementia. Kemper et al. report a prevalence of 12.5% for dementia in a German population aged 50 years and older after a first ischemic stroke [41]. We excluded patients with dementia because of their inability to consent which also might have contributed to the lesser agreement.

The agreement on chronic ischemic heart disease was moderate in our study. This result is consistent with those of other studies [11,13,17,32,34,36,40]. Some studies reported good to very good agreement on myocardial infarction or surgeries on the heart [9,13,14,32-34,37]. Presumably, the concordances were greater because the events are remembered better if surgical intervention took place, or if there was a myocardial infarction in the past.

For cardiac insufficiency we found only fair agreement, which were also seen in other studies [14,17,33]. Some studies reported moderate agreement [9,11,13,34]. Cardiac insufficiency belongs to those diseases which are difficult to communicate to the patients. The medical term is not very familiar to the patients. They probably know of the disease, but tend to call this a general heart disease. This makes it harder to distinguish from chronic ischemic heart disease.

We found a moderate agreement on cancer. A few studies reported the same results [9,40], while others demonstrated good to very good kappa coefficients [11,16,32-34,37]. This difference may be due to the data collection methods used for the cancer diagnosis variable in the MultiCare Cohort Study. In contrast to the patients, the GPs reported cancer nearly twice as often. Looking at the severity specified by the GPs, it is notable that the cancer is rated dormant in 34% of these cases. One reason for the moderate agreement might be that some patients did not report such a dormant cancer. We also saw a smaller percentage of patients who reported cancer when their GP did not. A further examination of this phenomenon is required.

The agreement for neuropathies had only a poor kappa coefficient and a slightly higher PA value. Neuropathies include many different symptoms with varying degrees of severity. This makes an agreement between GP and patient on the disease more difficult. Louie et al. found a moderate agreement for neurological complications in bone marrow transplantation survivors [40].

In this study, similar agreement values were found for dizziness: A kappa value of 14% and a PA of 25%. Dizziness is another disease which has no clear diagnostic criteria and who’s symptoms and causes vary greatly from patient to patient. Soto-Varela et al. validated a classification of peripheral vertigo by medical assessors. They saw a moderate agreement between the assessors regarding the accordance level [42]. Therefore, it is not surprising that the agreement between patient and GP was lower than that of medical experts.

Hemorrhoids and gynecological problems also had low agreement values. These two chronic conditions have the potential to be very shameful for some patients. In an elderly population, Sjahid et al. investigated the agreement levels between the drugs a patient presented during a patient interview and the drugs listed in that patient’s pharmacy records. They found the lowest kappa statistic for organo-heparinoids often used as ointments against hemorrhoids [43]. It is assumed, that some patients rather use over-the-counter products for their problem instead of talking to their GP. In addition, the interviewers in our project asked every patient directly about these diseases. This could explain the higher prevalence in the patient self-reported disease lists. Furthermore, the gynecological problems are principally treated by a gynecologist, so perhaps the patient feels no need to mention these to her GP.

### Agreement measures

The kappa coefficient is affected by two paradoxes. First, a high level of rater agreement can lead to a low kappa value. The formula for the kappa coefficient shows that the value of kappa depends on the level of chance agreement. Large chance agreement levels can lead to low kappa values despite observed high agreement levels. Second, imbalanced marginal totals affect the values of kappa. In case of an asymmetrical distribution of marginal totals, the chance agreement levels are lower which results in higher kappa values [44,45].

Therefore we calculated the proportions of specific agreement [25,46,47]. We saw large differences between the kappa coefficient and the PA for the chronic conditions: hypertension, lipid metabolism disorder, joint arthrosis and chronic low back pain. These diseases all had a high prevalence and the PA values were much higher than the kappa values. The differences could be caused by the paradoxes described above. Therefore, the proportions of specific agreement seem to be the better approach to observing agreement and are more informative for clinicians as previously described by de Vet and colleagues [46].

It is further noted that the prevalence differences between the reported diagnosis groups should be taken into account when interpreting the results. Missing agreement can be caused by such factors as systematic differences between the raters, recall bias or chance. If this absolute error rate is the same for all chronic conditions, the relative random error for low prevalent diseases would be higher than for highly prevalent diseases. Hence, poor agreement for diseases with low prevalence should be registered with caution.

### Patient characteristics associated with agreement

Our analyses of the associations of patient characteristics with agreement levels show that women agreed positively with their GPs on the diagnoses: osteoporosis, thyroid dysfunction, rheumatoid arthritis/chronic polyarthritis, varicosis, intestinal diverticulosis, joint arthrosis, chronic low back pain, chronic cholecystitis/gallstones and dizziness. The women’s odds ratios for positive agreement were lower for chronic ischemic heart disease, atherosclerosis/PAOD, renal insufficiency, hyperuricemia/gout, cerebral ischemia/chronic stroke, diabetes mellitus, cardiac arrhythmias, cancers, cardiac insufficiency and neuropathies. Englert et al. reported an association of the male gender with the over-reporting of myocardial infarction, stroke, hypertension and cardiac arrhythmias in a German study population of patients with hypercholesterolemia [17]. Kriegsman et al. reported this association for stroke as well [16]. This result reflects the gender-specific diseases. Cardio vascular diseases are more frequent in men and are, respectively, attributed rather to the men than to women. Furthermore, men may pay greater attention to male-specific diseases and women to female-specific diseases. As mentioned above, this might also be an effect of the diseases’ prevalences, as gender-specific differences in prevalence might also affect gender-specific agreement proportions.

We saw a negative association between positive agreement and increasing age in seven diagnosis groups. Other studies were able to show this as well e.g. for diabetes [13,14,16]. Whereas increasing age was also associated with better agreement for eight diagnosis groups. For cardiac insufficiency other studies reported lower associations between agreement and older age [13], higher associations between disagreement and increasing age [14] or saw no effect for age at all [34]. A higher prevalence of cardiac insufficiency in the older age group (75 years and more) in our cohort could possibly be a reason for better agreement. The results of other studies also varied for high blood pressure. Some saw no association between age and agreement [13,34], others reported an association between increasing age and poorer agreement [17,35] and others described more accurate self-reports for older hypertensive respondents [10]. Overall, the results on the association between agreement and age indicate that the agreement is higher for diseases associated with older age (e.g. cardiac insufficiency or renal insufficiency) or lower for diseases associated with lesser age (e.g. lipid metabolism disorders). Furthermore, this might be an effect of prevalence differences as already described.

For severe vision reduction, osteoporosis and thyroid dysfunction, a lower association to positive agreement was identified in patients with a low education level. It is assumed that patients with a higher education manage their medical records better. Rather surprisingly, we saw a lower odds ratio for positive agreement on atherosclerosis/PAOD in patients with higher education. This also might be an effect of prevalence considering that, in our cohort, the prevalence for atherosclerosis/PAOD is half as high in patients with higher level education as in patients with lower level education.

For asthma/COPD and diabetes mellitus the odds ratios for a positive agreement decreased with increasing income. Leikauf and Federman reported an association between low household incomes and fewer reports of asthma for inner-city seniors [35]. For cerebral ischemia/chronic stroke the odds ratio for positive agreement was higher with increasing income. Okura et al. found an association between higher education levels and better agreement for stroke in their unadjusted, logistic regression model [13]. Further investigations on this trend are required, especially for multimorbid older patients.

There was an association with the disease count for almost all examined diagnoses groups. The odds ratios for positive agreement increased with each additional disease. This may be an effect of a higher prevalence of the examined diseases in persons with more chronic conditions as reported by Schäfer [48]. Merkin et al. reported a positive association between reported congestive heart failure and the presence of additional chronic diseases [34]. Other studies described the opposite effect: Comorbidity results in poorer agreement [11,13]. It is possible that the patients in the MultiCare cohort differ from other studies regarding this effect, because all patients have at least three chronic conditions.

We saw a negative association of depression with the concordance of patient self-reported and general practitioner reported thyroid dysfunction. Corser et al. found this as well, but for other diseases (cerebrovaskular disease, chronic pulmonary disease, cancer and diabetes) [14]. For atherosclerosis/PAOD, dizziness and neuropathies, the analysis even revealed an increased probability for a positive agreement concerning depression. However, the sample size of patients in the MultiCare Cohort Study with a positive statement and depression (GDS > 6 points) was small (n = 119), which may result in low statistical power.

Patients with a higher score on the EQ visual analogue scale had lower odds ratios for positive agreement on ten chronic diseases. Most of these diseases are known for their high burden of pain or physical limitations, which are associated with a lower quality of life such as chronic low back pain, rheumatoid arthritis/chronic polyarthritis or asthma/COPD. Leikauf and Federman saw an association between poor general health in a patient and the accuracy of his/her self-report of asthma [35]. Patients with a lower score on the EQ visual analogue scale may belong to the severe cases. These cases might be better known by the general practitioners, and patients with a lower quality of life might be more motivated to inform themselves about their diseases. However, it should be noted that the effect sizes for this association were not very high.

Patients with a nursing care dependency had higher odds ratios for positive agreement on cancer, cerebral ischemia/chronic stroke and cardiac insufficiency. For patients with cancer, stroke and/or cardiac diseases, Kriegsman et al. described high odds ratios of patients over-reporting mobility impairments [16]. Regarding stroke, the relationship is obvious, since this often leads to dependency. Moreover, it can be assumed that due to the increased care burden in a patient with a nursing care dependency, the GP’s record keeping is more accurate. In a retrospective cross-sectional study, Erler et al. examined the agreement of patients’ medical records from German GP practices with claims-based diagnoses and observed an over-reporting of permanent chronic conditions. Non-severe diagnoses, on the other hand, which are frequently encountered in GP practices, were under-reported [49].

### Strengths and weaknesses

This is one of the few analyses of agreement which was performed with a large number of chronic conditions. Most studies considered fewer diseases. Furthermore, this study compared GP reports collected in personal interviews, where many other studies simply took medical records as a source for the physicians’ statements [9,12-14,32-37,50,51]. Few studies involved GPs in their analysis of concordance [15-17]. We assume that personal interviews have a better validity than an analysis of medical records because diagnoses are often only used to justify insurance claims and, therefore, might not be mentioned in the records if no intervention is necessary. The personal interviews by trained interviewers, with both GPs and their patients, enabled a particularly precise survey of chronic diseases. Missing information in the medical records or incomplete questionnaires have probably been avoided in many cases.

The generalizability of the MultiCare Cohort Study could be affected by our criteria for exclusion at baseline. We excluded patients with dementia because of their inability to consent as well as patients residing in a nursing home. Our recruitment only took place in larger German cities, so that rural areas were not included in our study. Nevertheless, our study is representative of an older, urban, multimorbid cohort in primary care [19]. We may have overestimated the validity of GP diagnoses, because the doctors who participated in the survey may have been more motivated than those who didn't participate. For this reason the agreement of GP and patient diagnoses could be lower in reality than as was presented in our study.

We found almost no inconsistencies in the dates of the interviews. In some cases the GP interview may have taken place after the patient interview so that, in the meantime, a new diagnosis could have been made, which was not yet known by the patient at the time of his interview. However, this related to an average of less than 1% of all cases.

Some of the diagnoses groups names in the patient interview were partially different than in the GP interview. We had to adapt the names of the diagnosis groups for the patient interview to a more patient-friendly language. This could have led to lower agreement values. For example, in patient interviews, cardiac insufficiency was named “weakness of the heart, which can cause fluid retention in the legs or lungs” because few patients understand the specialized term. Other studies reported this problem as well [11,17,33,35].

Assuming that patients are not aware of their disease if they don’t give an answer (cases we defined as missing values), we converted these missing values in the patients’ self-reports to a “no disease” report. Giving no answer could have several reasons, e.g. an interviewer mistake or vague statements on behalf of the patients. Nevertheless, this affects an average of only 0.13% of the cases. The maximum amount of missing self-reported diagnoses was found for rheumatoid arthritis/chronic polyarthritis (0.3%).

Furthermore, a multitude of procedures for the prevention of insufficient data quality, the detection of inaccurate or incomplete data and actions to improve the data’s quality were performed (e.g. user reliability trainings, automatic plausibility and integrity checks and data error reports to the collaborating centers).

We were able to obtain a high participation rate of 46%. The non-responder analysis found that there was a slight selection bias considering the younger age of the participants, but no bias regarding gender or 93% of the diagnosis groups. We recruited participants according to the practices’ chart registry and not through a waiting-room process. Therefore we should have no problems regarding the overestimation of conditions that lead to greater heath care utilization [19].

As a further strength, we calculated multivariate analyses in order to adjust for possible confounding.

## Conclusions

The data corresponds to the reality of primary care. Diseases with good agreement are easier to diagnose and often have established clinical guidelines (e.g. diabetes mellitus). Diseases with poor agreement are more difficult to diagnose (e.g. dizziness or neuropathies) and they are more difficult to communicate.

For multimorbidity research and the development of clinical guidelines, it is important to know which chronic diseases have a high disagreement between patient and physician reports. GPs should pay special attention to these chronic conditions. The analysis also shows that different patient characteristics have an impact on the quality of the agreement.

Further research is needed to identify more reasons for disagreement. In addition to the patient characteristics, there are certainly other reasons for a lack of agreement between the GP and his patient. Especially the low agreement on cancer or the association of increasing income and agreement for stroke should be considered in more detail. GPs and patients should possibly become more involved in this research topic (e.g. via qualitative research). Further studies may help to identify types of patients, who have particularly low agreement levels. A targeted communication with these patients may improve the patients’ understanding of their illnesses and increase the GP’s level of information about the patient. This assumption should be examined in further studies as well as the consequences of disagreement for health care. The results from this study might be useful in guiding the development of clinical guidelines and thus optimizing health care.

## Competing interests

The authors declare that they have no competing interests.

## Authors’ contributions

HvdB, IS, HH, KW, MS and BW conceived and designed the study. BW and GS prepared the data for analysis. HH and GS analyzed the data. HH drafted the manuscript. SRH, JG, SW, JJP, HHK, HB, AF and SH participated in the study design and implementation. All authors read and approved the final manuscript.

## Pre-publication history

The pre-publication history for this paper can be accessed here:

http://www.biomedcentral.com/1471-2296/15/39/prepub

## Supplementary Material

Additional file 1 Selection process of diagnosis groups included in the MultiCare Cohort Study. Click here for file
