# Supplementary material for: Agreement between self-reported and general practitioner-reported chronic conditions among multimorbid patients in primary care - results of the MultiCare Cohort Study
Source: BMC Fam Pract. 2014 Mar 1;15:39. doi: 10.1186/1471-2296-15-39 (PMC3946039; doi:10.1186/1471-2296-15-39)
Supplement: Additional file 1 — Selection process of diagnosis groups included in the MultiCare Cohort Study. [file 1471-2296-15-39-S1.pdf]

Additional file 1 – Selection process of diagnosis groups included in the MultiCare Cohort Study

No	**complete list** (46 diagnosis groups)	**GP interview baseline list** (38 diagnosis groups)	**patient interview baseline list** (32 diagnosis groups)	**selection for logistic regression** (26 diagnosis groups)
1	Hypertension	Hypertension	Hypertension	Hypertension
2	Lipid metabolism disorders	Lipid metabolism disorders	Lipid metabolism disorders	Lipid metabolism disorders
3	Chronic low back pain	Chronic low back pain	Chronic low back pain	Chronic low back pain
4	Severe vision reduction	Severe vision reduction	Severe vision reduction	Severe vision reduction
5	Joint arthrosis	Joint arthrosis	Joint arthrosis	Joint arthrosis
6	Diabetes mellitus	Diabetes mellitus	Diabetes mellitus	Diabetes mellitus
7	Chronic ischemic heart disease	Chronic ischemic heart disease	Chronic ischemic heart disease	Chronic ischemic heart disease
8	Thyroid dysfunction	Thyroid dysfunction	Thyroid dysfunction	Thyroid dysfunction
9	Cardiac arrhythmias	Cardiac arrhythmias	Cardiac arrhythmias	Cardiac arrhythmias
10	Obesity	Obesity		
11	Hyperuricemia/Gout	Hyperuricemia/Gout	Hyperuricemia/Gout	Hyperuricemia/Gout
12	Prostatic hyperplasia	Prostatic hyperplasia	Prostatic hyperplasia	Prostatic hyperplasia
13	Lower limb varicosis	Lower limb varicosis	Lower limb varicosis	Lower limb varicosis
14	Liver diseases	Liver diseases		
15	Depression	Depression		
16	Asthma/COPD	Asthma/COPD	Asthma/COPD	Asthma/COPD
17	Gynecological problems	Gynecological problems	Gynecological problems	
18	Atherosclerosis/PAOD	Atherosclerosis/PAOD	Atherosclerosis/PAOD	Atherosclerosis/PAOD
19	Osteoporosis	Osteoporosis	Osteoporosis	Osteoporosis
20	Renal insufficiency	Renal insufficiency	Renal insufficiency	Renal insufficiency
21	Cerebral ischemia/Chronic stroke	Cerebral ischemia/Chronic stroke	Cerebral ischemia/Chronic stroke	Cerebral ischemia/Chronic stroke
22	Cardiac insufficiency	Cardiac insufficiency	Cardiac insufficiency	Cardiac insufficiency
23	Severe hearing loss	Severe hearing loss		
24	Chronic cholecystitis/Gallstones	Chronic cholecystitis/Gallstones	Chronic cholecystitis/Gallstones	Chronic cholecystitis/Gallstones
25	Somatoform disorders	Somatoform disorders		
26	Hemorrhoids	Hemorrhoids	Hemorrhoids	Hemorrhoids
27	Intestinal diverticulosis	Intestinal diverticulosis	Intestinal diverticulosis	Intestinal diverticulosis
28	Rheumatoid arthritis/Chronic polyarthritis	Rheumatoid arthritis/Chronic polyarthritis	Rheumatoid arthritis/Chronic polyarthritis	Rheumatoid arthritis/Chronic polyarthritis
29	Cardiac valve disorders	Cardiac valve disorders	Cardiac valve disorders	Cardiac valve disorders
30	Neuropathies	Neuropathies	Neuropathies	Neuropathies
31	Dizziness	Dizziness	Dizziness	Dizziness
32	Dementias			
33	Urinary incontinence	Urinary incontinence		
34	Urinary tract calculi	Urinary tract calculi	Urinary tract calculi	
35	Anemias	Anemias	Anemias	
36	Anxiety	Anxiety		
37	Psoriasis	Psoriasis	Psoriasis	
38	Migraine/chronic headache	Migraine/chronic headache	Migraine/chronic headache	
39	Parkinson’s disease	Parkinson’s disease	Parkinson’s disease	

No	**complete list** (46 diagnosis groups)	**GP interview baseline list** (38 diagnosis groups)	**patient interview baseline list** (32 diagnosis groups)	**selection for logistic regression** (26 diagnosis groups)
40	Cancers	Cancers	Cancers	Cancers
41	Allergies			
42	Chronic gastritis/GERD			
43	Sexual dysfunction			
44	Insomnia			
45	Tobacco abuse			
46	Hypotension			

COPD: chronic obstructive pulmonary disease, PAOD: peripheral arterial occlusive disease
